# Clinical, Angiographic, and Procedural Characteristics of Patients Undergoing Percutaneous Coronary Intervention: A Single-Center Study From Northeast India

**DOI:** 10.7759/cureus.99301

**Published:** 2025-12-15

**Authors:** Kumar Pankaj Prabhat, Pranab J Bhattacharyya

**Affiliations:** 1 Cardiology, Narayan Medical College and Hospital, Sasaram, IND; 2 Cardiology, Gauhati Medical College and Hospital, Guwahati, IND

**Keywords:** angiographic profile, coronary artery disease, outcomes, percutaneous coronary interventions, risk factors

## Abstract

Objective: This study aims to evaluate the clinical, angiographic, and procedural characteristics, as well as short-term outcomes, of patients undergoing percutaneous coronary intervention (PCI) at a tertiary care center.

Materials and methods: A prospective observational study was conducted at Gauhati Medical College and Hospital, Guwahati, Assam, between March 2020 and December 2021, enrolling 150 patients undergoing PCI. Baseline demographic details, risk factors, clinical presentations, echocardiographic findings, angiographic profiles, procedural characteristics, and outcomes were recorded. Statistical analyses were performed using SPSS Statistics version 21.0 (IBM Corp. Released 2012. IBM SPSS Statistics for Windows, Version 21.0. Armonk, NY: IBM Corp.).

Results: The mean age of the study population was 55 ± 12 years, with 135 (90%) being male. Hypertension (93 (62%)) and dyslipidemia (86 (57%)) were the most common risk factors. Acute coronary syndrome (ACS) with ST-elevation myocardial infarction was the most frequent presentation (107 (71.33%)). Femoral access was used in the majority of procedures (146 (97.33%)), and elective PCI was performed in 115 (76.77%) of patients. Single-vessel disease was observed in 79 (53.33%) cases, and significant involvement of the left anterior descending (LAD) artery was noted in 109 (72.67%) cases. Type B lesions were the most common in 113 (75.33%) patients. Overall, one patient (0.67%) died during hospitalization, while 149 (99.33%) were discharged stable. Procedural complications occurred in 27 (18%) of patients, with slow flow being the most frequent (10 (6.67%)).

Conclusions: This study highlights a high burden of risk factors and ACS presentations among PCI patients in Northeast India, with LAD being the most commonly involved artery. PCI was safe and effective with high success and low complication rates. Broader studies are needed to validate these findings.

## Introduction

Coronary artery disease (CAD) continues to be a leading cause of morbidity and mortality globally, contributing significantly to the burden of cardiovascular diseases, particularly in developing countries [1]. In India, the burden of CAD has been rising steadily over the past few decades, with notable regional variations in clinical presentation, risk factors, and outcomes [2]. Percutaneous coronary intervention (PCI) has emerged as a cornerstone in the management of CAD, offering rapid revascularization and improved clinical outcomes across a broad spectrum of coronary presentations, ranging from chronic stable angina to acute coronary syndrome (ACS) [3].

Understanding the clinical profiles, angiographic characteristics, procedural patterns, and short-term outcomes of patients undergoing PCI is critical for optimizing patient care, especially in regions with diverse populations and healthcare access disparities. While a few studies have previously reported on PCI outcomes from the northeastern region of India [4,5], comprehensive data evaluating the clinical, angiographic, and procedural aspects of PCI in this region remain limited.

The present study was undertaken to address this gap by exploring the clinical profiles of patients with CAD undergoing PCI in a tertiary care center in Northeast India. Specifically, the study aimed to analyze risk factors, patterns of clinical presentation, angiographic findings, indications for PCI, patterns of stent use, procedural success rates, and adverse outcomes during hospitalization and at 30-day follow-up. By providing region-specific insights, this study aims to enhance understanding of CAD management among the northeastern Indian population and contribute to the growing body of evidence on PCI outcomes in real-world settings.

## Materials and methods

Study design

This hospital-based, prospective observational study was conducted in the Department of Cardiology, Gauhati Medical College and Hospital, Guwahati, Assam, from March 2020 to December 2021.

Ethical considerations

Ethical clearance was obtained from the Institutional Ethics Committee of Gauhati Medical College and Hospital (approval number: 190/2007/Pt II/MAR-2020/39, approval date: 04.06.2020) prior to initiating the study. Written informed consent was taken from all participants. Patient management was done according to the American Heart Association (AHA) and European Society of Cardiology (ESC) guidelines.

Study population

**Figure 1:**
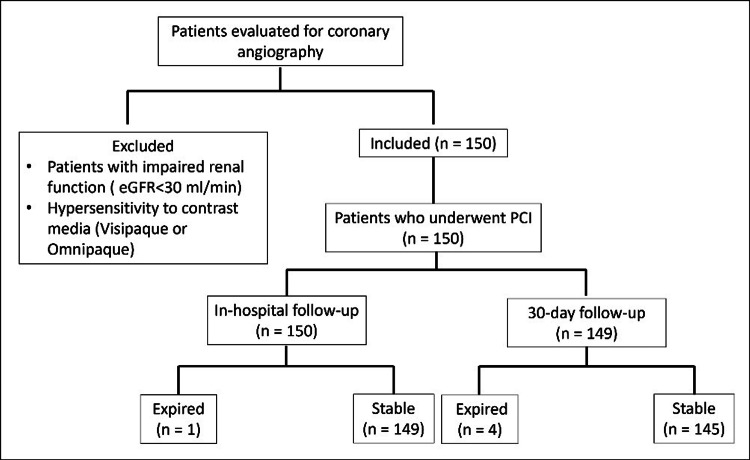
Study flow diagram eGFR: estimated glomerular filtration rate, PCI: percutaneous coronary intervention

Individuals presenting with CAD were prospectively enrolled based on clinical history, 12-lead electrocardiogram (ECG) findings, biochemical markers, or noninvasive evaluations such as treadmill testing or echocardiography, provided they subsequently underwent PCI. Patients were included regardless of their clinical presentation, encompassing stable angina, unstable angina (UA), ST-elevation myocardial infarction (STEMI), and non-ST-elevation myocardial infarction (NSTEMI). Inclusion was limited to those undergoing coronary angiography followed by PCI for either chronic coronary syndrome or ACS. Patients with impaired renal function (estimated glomerular filtration rate (eGFR) <30 ml/min), known hypersensitivity to contrast media (Visipaque or Omnipaque), or those unwilling to provide informed consent were excluded (Figure 1).

Data collection

Demographic data, clinical details, and atherosclerotic risk factors were recorded for all participants. The following risk factors were defined and documented:

Smoking was defined as regular smoking of one or more cigarettes per day for over six months or cessation within the last two years. Diabetes mellitus was diagnosed based on fasting plasma glucose ≥126 mg/dL, HbA1c ≥6.5%, random blood glucose >200 mg/dL with symptoms, or ongoing anti-diabetic therapy [6]. Hypertension was defined as blood pressure ≥140/90 mmHg on two separate occasions or current use of antihypertensive medications [7].

Dyslipidemia was defined by the presence of LDL-C levels >130 mg/dL (3.4 mmol/L) in the general population and >70 mg/dL (1.8 mmol/L) in individuals with cardiovascular disease, HDL cholesterol >40 mg/dL in males and >50 mg/dL in females, and triglyceride levels >150 mg/dL (1.7 mmol/L) [8,9]. Obesity was defined as BMI ≥23 kg/m² [10].

Angiographic and procedural details

Coronary angiographic assessment included the site, severity, type, and extent of lesions, as well as the number of vessels involved. Key definitions included the following:

Significant LMCA disease was defined as >50% stenosis. CAD was classified as single-vessel disease (SVD), double-vessel disease (DVD), or triple-vessel disease (TVD). Lesion complexity was graded according to the American College of Cardiology/American Heart Association (ACC/AHA) guidelines and further evaluated using the SYNTAX score (calculated via syntaxscore2020.com)

Lesions with ≥50% stenosis in LMCA or ≥70% in other vessels (reference vessel diameter ≥2.25 mm) were treated with stenting. Severe stenosis in vessels <2.25 mm was either left untreated or managed with plain old balloon angioplasty (POBA) at the operator's discretion.

Procedural details documented included the vascular access route, site, and number of lesions treated; stent size and number; use of adjunctive devices; procedural success; and in-hospital complications. All patients were followed up for one month to evaluate early procedural outcomes.

Outcomes

The primary outcome of the study was the procedural success and short-term (30-day) survival following PCI. The secondary outcomes included assessment of patient profiles, cardiovascular risk factors, clinical presentation patterns, angiographic findings, procedural characteristics, complications, and short-term (in-hospital and 30-day) adverse events.

Operational definitions

Key clinical and angiographic terms are defined as follows: Stable angina was defined as typical or atypical chest pain with noninvasive evidence of ischemia. While myocardial infarction (MI) was considered based on ischemic chest pain >30 minutes, ECG changes, or a positive troponin assay, it was further classified into STEMI and NSTEMI. UA is considered ischemic chest pain with ECG ST depression but negative troponins. Lesion Types (A, B, C), classified based on complexity, tortuosity, calcification, and occlusion duration according to ACC/AHA classification.

Other definitions are as follows: Coronary artery segments were defined as the left main coronary artery, which is considered a segment and a territory of its own. Proximal segments comprised the proximal parts of the left anterior descending (LAD), the left circumflex (LCX), and the right coronary artery (RCA).

Ostial stenosis was defined as a stenosis that is classified as “ostial” when the origin of the lesion was within 3 mm of the vessel origin involved. A thrombus was defined as a discrete, intraluminal filling defect with defined borders and largely separated from the adjacent vessel wall. Contrast staining might or might not be present.

Tortuosity was defined as stenosis distal to two bends >75, which is considered moderately tortuous, and those distal to three or more bends >75 are considered excessively tortuous. Bifurcation stenosis was defined as stenosis involving the parent and daughter branch if a medium or large branch (>2.5 mm) originated within the stenosis and if the side branch was surrounded by stenotic portions of the lesion to be dilated.

Calcification is defined as calcification recorded if readily apparent densities are seen within the apparent vascular wall of the artery at the site of the stenosis. Chronic total occlusion was defined as a total occlusion (thrombolysis in myocardial infarction (TIMI) flow grade 0), judged to be three months in duration based on clinical and angiographic findings, and is considered a chronic total occlusion.

Eccentric stenosis is classified as eccentric when one of its luminal edges is in the outer one-quarter of the apparent normal lumen. Irregular contour, defined as a stenosis, is classified as having irregular contour if the vascular margin is rough or has a “saw tooth” appearance.

Procedural success was defined as residual stenosis <10% in stented segments or <50% in balloon angioplasty segments, with TIMI III flow and absence of significant clinical complications. Procedural complications are defined as death, MI, emergency coronary artery bypass grafting, stroke, vascular complications, bleeding, coronary perforation, stent thrombosis, arrhythmias requiring intervention, and contrast-induced acute kidney injury, based on contemporary guideline definitions.

Sample size calculation

A total of 180 subjects undergoing PCI were planned for enrollment, with an anticipated 20% dropout rate, to ensure that at least 150 evaluable patients would complete the study. We estimated the minimum sample using the single-population proportion formula:

$n = \frac{Z_{1-\alpha/2}^2 \, p(1-p)}{d^2}$

The calculation was based on the prevalence of hypertension (48.4%) reported in the Kerala ACS Registry [11], with Z = 1.96, an absolute precision (d) of 0.08, and α = 0.05. This yielded a minimum required sample of 150 subjects. The achieved enrollment of 150 patients therefore provided adequate precision to estimate major clinical and angiographic characteristics in the study population.

Statistical analysis

Statistical analysis was performed using SPSS Statistics version 21.0 (IBM Corp. Released 2012. IBM SPSS Statistics for Windows, Version 21.0. Armonk, NY: IBM Corp.). Descriptive statistics were used to summarize the data, with means and standard deviations reported for continuous variables and frequencies and percentages for categorical variables. Inferential statistics were applied as appropriate: independent sample t-tests or Mann-Whitney U tests were used to compare two groups. Chi-square or Fisher's exact tests were used to analyze categorical variables. A p-value < 0.05 was considered statistically significant.

## Results

The study included 150 patients who underwent PCI. The mean ± SD age of the study participants was 55 ± 12 years, with females having a significantly higher mean age (63 ± 9 years) than males (54 ± 12 years). The majority of patients (53 (35.3%)) were in the 55-64 years age group, followed by 24% in the 45-54 years age group. Notably, 28 (18.67%) of patients were younger than 45 years, reflecting a significant burden of premature coronary artery disease. Patients aged 65-74 years accounted for 28 (18.67%), while only 4 (2.67%) were aged 75 years and above. The majority of patients were male (135 (90%)). Common cardiovascular risk factors included hypertension (93 (62.00%)), dyslipidemia (86 (57.33%)), smoking (77 (50.67%)), diabetes mellitus (70 (47.33%)), and obesity (35 (23.33%)). Among the study participants, smoking was significantly more prevalent in males compared to females (75 (54.81%) vs. 2 (13.33%)). However, the prevalence of obesity and dyslipidemia was significantly higher in females compared to males (p = 0.032 and p = 0.046, respectively). The mean systolic and diastolic blood pressures were 132 ± 19 mmHg and 79 ± 8 mmHg, respectively. The average LDL cholesterol was 132 ± 41 mg/dL, and the mean HbA1c was 6 ± 1% (Table 1).

**Table 1:** Baseline demographic and clinical characteristics Data presented as mean (SD), unless otherwise specified. DBP: diastolic blood pressure, FBS: fasting blood sugar, HbA1c: glycated hemoglobin A1c, HDL: high-density lipoprotein cholesterol, LDL: low-density lipoprotein cholesterol, SBP: systolic blood pressure, TG: triglycerides, SD: standard deviation

Parameters	Female (n = 15)	Male (n = 135)	Total (N = 150)	p-value
Age (years)	63.00 ± 9.00	54.00 ± 12.00	55.00 ± 12.00	-
Age groups (years), n (%)				
25-34	-	8 (5.93)	8 (5.33)	-
35-44	1 (6.67)	19 (14.07)	20 (13.33)	-
45-54	-	36 (26.67)	36 (24.00)	-
55-64	6 (40.00)	47 (34.81)	53 (35.33)	-
65-74	7 (46.67)	21 (15.56)	28 (18.67)	-
75-84	1 (6.67)	3 (2.22)	4 (2.67)	-
85-94	-	1 (0.74)	1 (0.67)	-
Risk factors, n (%)				
Diabetes	9 (60.00)	62 (45.93)	71 (47.33)	0.321
Hypertension	9 (60)	84 (62.22)	93 (62.00)	0.512
Smoking	2 (13.33)	74 (54.81)	77 (50.67)	0.004
Obesity	8 (53.33)	27 (20.00)	35 (23.33)	0.032
Dyslipidemia	11 (73.33)	75 (55.56)	86 (57.33)	0.046
Biochemical parameters				
Pulse rate (bpm)	-	-	73.00 ± 8.00	-
SBP (mmHg)	-	-	132.00 ± 19.00	-
DBP (mmHg)	-	-	79.00 ± 8.00	-
LDL (mg/dL)	-	-	132.00 ± 41.00	-
TG (mg/dL)	-	-	216.00 ± 85.00	-
HDL (mg/dL)	-	-	37.00 ± 8.00	-
FBS (mg/dL)	-	-	120.00 ± 50.00	-
HbA1c (%)	-	-	6.00 ± 1.00	-

Regarding clinical presentation, 107 (71.33%) patients presented with STEMI, 17 (11.33%) with NSTEMI, four (2.67%) with UA, and 23 (15.33%) with chronic stable angina. Thrombolysis had been administered in 35 (23.49%) of patients before PCI. ECG analysis revealed ST elevation in 100 (66.67%) cases; 12 (8%) showed wave inversion, and 123 (82%) had positive troponin T levels (Table 2).

**Table 2:** Clinical presentation and ECG findings Data presented as n (%). ACS: acute coronary syndrome, CHB: complete heart block, CSA: chronic stable angina, ECG: electrocardiogram, LBBB: left bundle branch block, NSTEMI: non-ST-elevation myocardial infarction, QS pattern: pathological Q wave and secondary S wave pattern (suggestive of infarction), ST elevation: ST segment elevation, ST depression: ST segment depression, STEMI: ST-elevation myocardial infarction, TROP T: troponin T, UA: unstable angina

Findings	Total (N = 150)
Diagnosis group	
ACS/STEMI	107 (71.33)
ACS/NSTEMI	17 (11.33)
UA	4 (2.67)
CSA	22 (14.67)
Thrombolysis status	
Yes	35 (23.49)
No	115 (76.51)
ECG group	
Normal	3 (2.00)
ST elevation	100 (66.67)
ST depression	12 (8.00)
T wave inversion	27 (18.00)
LBBB	4 (2.67)
QS pattern	2 (1.33)
CHB	2 (1.33)
TROP T status	
Negative	27 (18.00)
Positive	123 (82.00)

The TROP T was positive in 13 (86.66%) of females and 109 (81.48%) of males, with a statistically significant difference (p = 0.0001). Most of the patients with hypertension had ACS/STEMI (65 (60.75%)), CSA (14 (63.64%)), ACS/NSTEMI (12 (70.69%)), and UA (2 (50%)) (Table 3).

**Table 3:** Distribution of hypertension and smoking among patients Data presented as n (%). ACS: acute coronary syndrome, CSA: chronic stable angina, NSTEMI: non-ST-elevation myocardial infarction, ST elevation: ST segment elevation, ST depression: ST segment depression, STEMI: ST-elevation myocardial infarction, UA: unstable angina

Parameters	ACS/NSTEMI (n = 17)	ACS/STEMI (n = 107)	CSA (n = 22)	UA (n = 4)
Hypertension (n = 93)	12 (70.69)	65 (60.75)	14 (63.64)	2 (50.00)
Smoking (n = 76)	8 (47.06)	58 (54.21)	9 (40.91)	2 (50.00)

Echocardiography demonstrated regional wall-motion abnormalities in 131 (87.33%) patients and global hypokinesia in six (4%) patients. Mild, moderate, and severe left ventricular systolic dysfunction were present in 97 (64.67%), 25 (16.67%), and 10 (6.67%) of patients, respectively, while 18 (12%) had normal systolic function (Table 4).

**Table 4:** Echocardiographic and left ventricular systolic function assessment Data presented as n (%). ECHO: echocardiography, LVSD: left ventricular systolic dysfunction, RWMA: regional wall motion abnormality

Characteristics	Total (N = 150)
ECHO findings	
Normal ECHO study	12 (8.00)
No RWMA	1 (0.67)
RWMA	131 (87.33)
Global hypokinesia	6 (4.00)
LVSD category	
Mild	97 (64.67)
Moderate	25 (16.67)
Severe	10 (6.67)
Normal	18 (12.00)

Femoral artery access was used in 146 (97.33%) of PCI procedures (Table 5). Drug-eluting stents (DES) were used in all cases. Elective PCI was performed in 115 (89.84%) cases, pharmaco-invasive PCI in 29 (22.66%), and primary PCI in four (3.13%) cases. The majority of patients (91 (62.33%)) received one stent, with smaller proportions receiving two (43 (29.45%)), three (11 (7.53%)), or four stents (1 (0.68%)).

**Table 5:** Procedural characteristics and interventions Data presented as n (%). PCI: percutaneous coronary intervention, POBA: plain old balloon angioplasty

Characteristics	Total (N = 150)
Approach of PCI	
Femoral	146 (97.33)
Radial	4 (2.67)
PCI	n = 148
Primary PCI	4 (3.13)
Elective PCI	115 (89.84)
Pharmaco-invasive PCI	29 (22.66)
POBA	2 (1.33)
No. of stents	n = 146
1	91 (62.33)
2	43 (29.45)
3	11 (7.53)
4	1 (0.68)

Angiographic findings showed that significant lesions most commonly involved the LAD artery (109 (72.67%)), followed by the RCA (68 (45.33%)) and LCX artery (50 (33.33%)). SVD was present in 79 (52.67%) of patients. Lesions were classified as type A in 69 (46.00%), type B in 113 (75.33%), and type C in 46 (30.67%) of cases (Table 6).

**Table 6:** Angiographic findings and lesion characteristics Data presented as n (%). DVD: double-vessel disease, LAD: left anterior descending artery, LCX: left circumflex artery, LM: left main coronary artery, RCA: right coronary artery, SVD: single-vessel disease, TVD: triple-vessel disease

Coronary angiographic profile	Total (N = 150)
Significant lesion	
LM (≥50%)	4 (2.67)
LAD (≥70%)	109 (72.67)
LCX (≥70%)	50 (33.33)
RCA (≥70%)	68 (45.33)
Type of lesion	
Type A	69 (46.00)
Type B	113 (75.33)
Type C	46 (30.67)
Number of vessels involved	
SVD	79 (52.67)
DVD	58 (38.67)
TVD	13 (8.67)

Among 150 patients, 135 (90%) had a SYNTAX score <22, 15 (10%) had scores between 22 and 32, while none had scores >32. All 79 (58.52%) SVD cases were in the <22 group, while 51 (53.33%) patients had DVD and five (3.70%) had TVD. Additionally, seven (46.67%) DVD patients and eight (53.33%) TVD patients had a SYNTAX score between 22 and 32. All procedures were performed on patients with a SYNTAX score <32. Patients with lower SYNTAX scores had better outcomes, while scores between 22 and 32 showed slightly poorer results, and lower SYNTAX scores were associated with better clinical outcomes (Table 7).

**Table 7:** SYNTAX score outcomes Data represented as n (%). DVD: double-vessel disease, SVD: single-vessel disease, TVD: triple-vessel disease

SYNTAX score	<22 (n = 135)	22-32 (n = 15)	>32 (n = 0)
SVD	79 (58.52)	-	-
DVD	51 (53.33)	7 (46.67)	-
TVD	5 (3.70)	8 (53.33)	-
In-hospital outcomes			
Expired	1 (0.74)	-	-
Stable	134 (99.26)	15 (100.00)	-

Procedural complications occurred in 27 (18%) patients, with the most frequent being slow flow (10 (6.67%)). Procedural success was achieved in 149 out of 150 patients (99.33%), characterized by restoration of TIMI III flow with minimal residual stenosis and a low in-hospital mortality rate of 0.67% due to arrhythmia with acute left ventricular failure. Only one patient had TIMI II flow, mainly due to complications such as slow flow/no-reflow. During short-term follow-up, four additional deaths were reported: drug defaulters, arrhythmia complications, and severe left ventricular failure. Half were likely due to stent thrombosis in high-risk patients who defaulted on dual antiplatelet therapy. Others had advanced heart failure. Overall, 85% of patients attended in-person follow-up within one month, while 15% were followed up by phone (Table 8).

**Table 8:** Procedural clinical outcomes Data presented as n (%). CHB: complete heart block, CIN: contrast-induced nephropathy, LVF: left ventricular failure, VT: ventricular tachycardia

Complication	Total (N = 150)
Procedural complications	n = 27
Acute LVF	2 (1.33)
VT	3 (2.00)
Air embolism	1 (0.67)
CHB	1 (0.67)
CIN	5 (3.33)
Side branch loss	2 (1.33)
Slow flow	10 (6.67)
Stent under expansion	1 (0.67)
Vasovagal	2 (1.33)
In-hospital outcomes	
Expired	1 (0.67)
Stable	149 (99.33)
1-month follow-up outcomes	n = 149
Expired	4 (2.68)
Stable	145 (97.31)

## Discussion

This prospective study analyzed the demographic, clinical, angiographic, and procedural characteristics of 150 patients undergoing PCI at Gauhati Medical College and Hospital, the largest government tertiary care center in Northeast India.

Compared with the National Interventional Council (NIC) 2017 data [12], in the present study, 18.67% of PCI procedures involved patients aged under 45 years, a notably higher proportion than the 12.24% reported in the NIC’s 2017 registry. Only 2.7% of patients were aged ≥75, far below the roughly 17% seen nationally, possibly reflecting a more conservative strategy during the initial phase of our cath lab. Additionally, the present study showed a more pronounced male predominance at 90%, versus 70% in the NIC data, which may reflect under-recognition of CAD in women and disparities in healthcare access. While NIC 2017 showed an increasing trend in female PCI volumes, our center still observed a disproportionately low representation of women, underscoring the need for greater awareness and equitable referral patterns.

Diabetes mellitus was more common among females (60%) than males (45.19%). This pattern mirrors observations in the Kerala ACS registry and in studies from Northeast India [4,11]. Hypertension was observed in 62% of patients, consistent with the Srinagar registry (74.8%) and other Indian studies [5,13]. Smoking, a major modifiable risk factor, was significantly more prevalent among males (55.6%) than females (13.3%) (p = 0.004), a finding echoed in the Srinagar registry, where 93.6% of male patients were smokers [13]. These findings underscore the need for targeted preventive strategies based on risk stratification.

A study by Gupta et al. reported that smokeless tobacco use is prevalent in countries across South and Southeast Asia, Africa, and Northern Europe. Users of smokeless tobacco show a higher prevalence of hypertension, fatal coronary heart disease, fatal stroke, and metabolic syndrome. Its use contributes to accelerated atherothrombosis, comparable to the effects of smoking [14]. Smokeless tobacco use has increasingly adverse effects on both the cardiovascular and respiratory systems, making it a significant public health concern [15]. Additionally, in the present study area, the majority of patients chew tobacco and consume betel nut, which may be one of the associated factors for the progression of CAD in these patients.

Approximately 82% of patients in this study presented with ACS, with STEMI being the most common subtype, accounting for 70.67% of cases. This is significantly higher than the 16.17% STEMI rate reported in the NIC 2017 data [12], reflecting a greater acute presentation burden in our region. NSTEMI and UA were observed in 2.67% of patients, which was comparatively lower than the NIC-reported rate of 25.8%. Chronic stable angina was seen in 15.33% of our cases, slightly lower than the 19.34% noted nationally.

Only 23.49% of STEMI patients received thrombolysis, often due to late arrival beyond the recommended window period. This rate is lower than in the CREATE registry (58.5%) and comparable to findings from other Indian studies [4,16]. These gaps highlight the need for public education, early referral systems, and improved ambulance availability in rural and hilly regions. Troponin T positivity was observed in 82% of the cohort, indicating a high prevalence of acute myocardial injury at presentation. These findings are comparable to other Indian datasets focused on PCI in acute settings [4,17]. Also, the higher troponin positivity among females may suggest delayed presentation or diagnostic variability across sexes.

ECG analysis revealed that ST elevation was the most frequent finding, present in 66.67% of patients, aligning with the high rate of STEMI diagnosis. Only 2% of patients had a normal ECG, suggesting that most patients presented with significant ischemic changes, emphasizing both the severity of cardiac presentations and the critical role of early ECG interpretation in guiding timely diagnosis and management of CAD. Echocardiography revealed regional wall motion abnormalities in 87% of patients, with global hypokinesia in 4%. Left ventricular systolic dysfunction was present in more than half of the patients, with moderate-to-severe dysfunction in 23%. These findings are consistent with the regional registry and underscore the burden of myocardial injury at presentation [13].

Elective PCI accounted for the majority of procedures in our study (76.66%), whereas primary PCI and pharmacoinvasive strategies were performed in only 2.7% and 19.3% of cases, respectively. The rate of primary PCI was substantially lower than the 13.74% reported in the NIC 2017 data [13], reflecting ongoing challenges such as delayed presentation, limited public awareness of ischemic symptoms, inadequate emergency transport systems, and underdeveloped STEMI referral networks. These findings underscore the need to strengthen early recognition, referral mechanisms, and infrastructure in resource-limited regions like Northeast India to ensure timely revascularization and improved outcomes.

Angiographic analysis revealed that the LAD artery was the most commonly involved vessel (72.67%). SVD was seen in 50% of patients, and type B lesions were the most frequent (75.33%). These findings align with previous regional studies and highlight a predominance of moderate-to-complex coronary anatomy in real-world PCI practice [5,13].

Timely primary PCI is critical in the management of STEMI. The interval from STEMI diagnosis to primary PCI in patients presenting directly to a tertiary care center should be within 60 minutes, with an acceptable delay of up to 120 minutes in select situations [18]. The present study was conducted at Gauhati Medical College and Hospital, the largest government tertiary care center in Northeast India. As the sole referral facility for a vast, geographically dispersed population, it experiences a consistently high patient influx that can strain existing infrastructure and limit the availability of emergency services, including timely PCI. One of the critical challenges in this setting was the limited availability of organized ambulatory services, which often results in significant delays in patient transport and triage. These delays hinder early hospital arrival and contribute to missed opportunities for reperfusion within the ‘golden hour,’ which is essential for optimal myocardial interventions and outcomes. Collectively, these systemic, logistical, and infrastructural barriers may underlie the increased mortality observed in this population with ACS.

Complications were observed in 18% of cases, including slow flow (6.67%), contrast-induced nephropathy (3.33%), and arrhythmias, all of which were managed successfully. These outcomes are comparable to studies by Sanghvi et al. and the Srinagar registry, which reported complication rates of 16-18% [13,19]. Short-term follow-up showed excellent outcomes, with 97.31% of patients alive and stable at one month. These results reflect outcomes reported in similar Indian PCI cohorts [20-22]. Providing intensive behavioral support interventions (individual face-to-face counselling, face-to-face group counselling, or telephone counselling) for smokeless tobacco users may benefit in reducing CVD progression.

The study has several limitations. It was conducted at a single center with a modest sample size, and the follow-up was limited to 30 days, precluding evaluation of long-term outcomes, including restenosis, target-lesion revascularization, and major adverse cardiovascular events. The study period coincided with the COVID-19 pandemic, which may have influenced patient presentations, referral patterns, and procedural volumes. Exclusion of patients with eGFR <30 mL/min and the small female cohort, as well as the single-center, largely single-operator practice, may restrict the generalizability of findings. Future multicenter studies with larger cohorts and extended follow-up are needed to validate these observations and address the limitations of the current study.

## Conclusions

This study provides valuable insights into the clinical, angiographic, and procedural profiles of patients undergoing PCI in Northeast India. A high burden of traditional risk factors, particularly among males and older females, was observed. Most patients had single-vessel, less complex disease, and procedural success was achieved in the majority of cases. Short-term mortality remained low, although periprocedural complications were not uncommon. These findings underscore the need to continue emphasizing early detection, risk factor management, and timely referral to improve outcomes in this population. Addressing these systemic challenges through public education, improved referral networks, and enhanced infrastructure may significantly improve outcomes in resource-limited settings. Longer-term follow-up studies are warranted to better evaluate the sustained safety and effectiveness of PCI in this regional context.
